# Salidroside ameliorated hypoxia‐induced tumorigenesis of BxPC‐3 cells via downregulating hypoxia‐inducible factor (HIF)‐1α and LOXL2

**DOI:** 10.1002/jcb.29000

**Published:** 2019-06-04

**Authors:** Xiaoping Chen, Yubin Kou, Yunsong Lu, Yumei Pu

**Affiliations:** ^1^ Department of Biliary and Pancreatic Surgery of Baoshan Branch Shuguang Hospital Affiliated to Shanghai University of Traditional Chinese Medicine Shanghai China; ^2^ Department of Hepatology Shanghai Skin Disease Hospital Shanghai China

**Keywords:** BxPC‐3 cells, HIF‐1α, LOXL2, pancreatic cancer, salidroside

## Abstract

Herein, we found that salidroside suppressed hypoxia‐inducible factor 1 alpha (HIF‐1α) and lysyl oxidase‐like protein 2 (LOXL2) within human pancreatic cancer BxPC‐3 cells cultured both under normoxia and hypoxia condition. To investigate the effect of salidroside on tumorigenesis of BxPC‐3 cells and whether HIF‐1α and LXCL2 were involved in this process, cells transfected with or without LOXL2 overexpression vector, were treated with 50 μg/mL of salidroside or 50 μM of KC7F2 (a HIF‐1α inhibitor) under hypoxia. Cell viability and invasion were assessed using CCK‐8 and Transwell chamber assay, respectively. Expression of E‐cadherin and matrix metalloproteinase 2/9 (MMP 2/9) was determined, by Western blot analysis, to assess cell mobility at molecular levels. We confirmed that hypoxia increased LOXL2 and induced tumorigenesis of BxPC‐3 cells, as evidenced by promoted cell proliferation and invasion, enhanced MMP2/9 while reduced E‐cadherin. Interestingly, hypoxia‐induced carcinogenesis was significantly retarded by both salidroside and KC7F2, however, enhanced with LOXL2 overexpression. Besides, salidroside and KC7F2 reduced LOXL2, and reversed the tumorigenesis of BxPC‐3 cells induced by LOXL2 overexpression. Given the inhibitory effect of salidroside on HIF‐1α expression, our data suggested that: (1) LOXL2 was the mechanism, whereby salidroside and KC7F2 showed inhibitory effect on cancer progression of BxPC‐3 cells; (2) salidroside exerted its anticancer effect, most likely, by a HIF‐1α/LOXL2 pathway. In conclusion, salidroside was a novel therapeutic drug in pancreatic cancer, and downregulation of HIF‐1α and LXCL2 was the underlying mechanism.

## 1 INTRODUCTION

Pancreatic cancer (PC) is a highly aggressive malignancy with chemotherapy‐resistant characteristic. 95% of PC is pancreatic ductal adenocarcinoma (PDA), which is the most lethal cancer with a 5‐year survival rate of stage IV merely 2% or less.^1^ Hypoxic microenvironment is commonly found in solid tumors of PC, which has been recognized as a major stimulus for angiogenesis, sustained PC cell growth and invasion, the induction of epithelial‐mesenchymal transition (EMT) process, and the insensitivity to radio‐ and chemo‐ therapies.^2^ Exploring novel agents or strategies based on hypoxia‐induced injury is necessary and urgent for PC blockade.

Hypoxia‐associated factor (HIF‐1α), a transcription factor in response to hypoxia, is strongly and specifically enhanced in PC. HIF‐1α is a master regulator of oncogenes or several key mechanisms that are closely related to PC carcinogenesis.^2^, ^3^ LOX family members are the target genes of HIF‐1α.^4^, ^5^ LOXL2, a member of LOX family, plays a role in PC treatment, and its downregulation attenuates EMT‐like process and inhibits invasiveness and metastasis of PC cell lines.^6^ Thus, exploring novel agents targeting HIF‐1α and LOXL2 may represent therapeutic strategies in PC prevention.

Phytochemicals can be used in adjuvant therapies in conventional anticancer treatment.^7^ Salidroside, a phenylpropanoid glycoside isolated from *Rhodiola rosea* L, possesses antioxidant and antihypoxic properties.^8^, ^9^ Currently, antitumor effect of salidroside has been proved in human breast cancer,^10^ fibrosarcoma,^11^ skin cancer,^12^ and renal cell carcinoma.^13^ Salidroside regulates the expression of HIF‐1α in hypoxia‐injured cardiomyocytes.^14^ However, whether salidroside plays a role in PC progression and whether HIF‐1α and LOXL2 were involved in this process remained largely unknown.

In our present study, BxPC‐3 cells, one of human PC cell lines, were stimulated with hypoxia. Cell proliferation and invasion were measured to assess tumorigenesis of BxPC‐3 cells. KC7F2 was used for intervening HIF‐1α effects according to reported studies.^15^, ^16^, ^17^ Besides, the effects of salidroside on in vivo tumor growth were also assessed in a mouse xenograft model. Our data suggested that salidroside can be used as a therapeutic agent in PC treatment, and downregulating HIF‐1α and LOXL2 was the underlying mechanisms.

## 2 MATERIALS AND METHODS

### 2.1 Cell culture

BxPc‐3 cells (American Type Culture Collection, Manassas, VA) grew in a medium containing 89% of Roswell Park Memorial Institute (RPMI)‐1640 (Hyclone), 10% of fetal bovine serum (FBS), and 1% mixing liquid of penicillin (100 U/mL, solarbio)/streptomycin (0.1 mg/mL, solarbio). In log‐phase growth, BxPc‐3 Cells were exposed to hypoxia (0.5% oxygen) and normoxia (21% oxygen), and maintained in 5% CO_2_ at 37°C for 2 days followed by serum‐starveling 1 day prior the further experiments. For cell invasion assay, cells grew in Dulbecco's modified Eagle's medium (DMEM) without FBS. In hypoxia studies, the choice of experimental conditions was supported in a reported study.^18^

### 2.2 Quantitative real‐time polymerase chain reaction (qRT‐PCR)

Total RNA from BxPc‐3 cells was extracted via trizol regent (1596‐026, Invitrogen), and reverse‐transcribed using RevertAid First Stand complementary DNA (cDNA) Synthesis kit (#K1622, Fermentas). Messenger RNA (mRNA) levels of LOX, LOXL1, LOXL2, LOXL3, LOXL4, HIF‐1α, E‐cadherin, MMP2, and MMP9 were quantified using a SYBR Green PCR Mix (Thermo, Shanghai, China) on ABI Prism 7300 SDS system (Applied Biosystem, Foster City, CA). Related primer sequences were listed in Table 1.

**Table 1:** Primers used in RT‐PCR analysis

Name	GenBank	Primer (5′‐3′)
*LOX*	NM_001178102.2, at 1931‐2133 position	Forward: AACATCATCCTGGGTTATTC; reverse: CTATTATGGCACATGGTTTC; 203 bp
*LOXL1*	NM_005576.3, at 3‐168 position	Forward: TCATTCAGAGTGGGAAAG; reverse: CGATGACAGCATTTCAAG; 166 bp
*LOXL2*	NM_002318.2, at 3528‐3772 position	Forward: CGTGTCTGTGTTTCCTTTG; reverse: ATGACTCCTGTTCCGTTAC; 245 bp
*LOXL2*	NM_033325.2, at 1158‐1286 position	Forward: GTGGAGGTGCTGAAGAATG; reverse: TAGTCTGGAGCCTGTGATG; 129 bp
*LOXL3*	NM_001289164.2, at 2053‐2172 position	Forward: CAACCGCACTCATCAGAC; reverse: GCCTGGGAGCAAAGATTC; 120 bp
*LOXL4*	NM_032211.6, at 2449‐2712 position	Forward: TGCCGATACACCAGATAC; reverse: GCAAGATTCAGGGATGAC; 264 bp
*HIF1α*	NM_001313919.1, at 1062‐1226 position	Forward: CAAGAAACCACCCATGAC; reverse: GGCTCATAACCCATCAAC; 165 bp
*E‐cadherin*	NM_009864.3, at 781‐1071 position	Forward: GACAGGCTGGCTGAAAGTG; reverse: TGGCTGACGATGGTGTAGG; 291 bp
*MMP2*	NM_008610.3, at 2162‐2285 position	Forward: TGGAATGCCATCCCTGATAAC; reverse: CAAACTTCACGCTCTTGAGAC; 124 bp
*MMP9*	NM_013599.4, at 2032‐2260 position	Forward: TCATTCGCGTGGATAAGGAG; reverse: CACGGTTGAAGCAAAGAAGG; 229 bp
*GAPDH*	NM_001256799.1, at 436‐653 position	Forward: AATCCCATCACCATCTTC; reverse: AGGCTGTTGTCATACTTC; 218 bp
*GAPDH*	NM_001289726.1, at 691‐887 position	Forward: CTGCCCAGAACATCATCC; reverse: CAGATGCCTGCTTCAC; 197 bp

Abbreviation: RT‐PCR, reverse‐transcription polymerase chain reaction.

### 2.3 Western blot analysis

Protein level in lysis supernatant of BxPc‐3 cells was determined by bicinchoninic acid (BCA) protein assay kit (Thermo), and 25 μg of which was separated on 10% to 15% of sodium dodecyl sulfate‐polyacrylamide gel electrophoresis (SDS‐PAGE). Electrophoretic pure of HIF‐1α, LOXL2, E‐cadherin, MMP2, and MMP9 were transferred onto nitrocellulose (NC) membranes (Millipore), and incubated with antibody against HIF‐1α (abcam, Ab216842), anti‐LOXL2 antibody (abcam, Ab96233), antibody against E‐cadherin (Cell Signaling Technology [CST], #14472), antibody against MMP2 (Abcam, Ab14311), antibody against MMP9 (Abcam, Ab137867), anti‐β‐actin antibody (Abcam, ab8226), and anti‐GAPDH antibody (CST, #5174) at 4°C overnight followed by horseradish peroxidase‐conjugated antibodies (Beyotime, A0208, Shanghai, China) for another 1 hour at 25°C. Immunoreactive bands were qualified by enhanced chemiluminescence (ECL) system (GE Healthcare/Amersham Biosciences). We chose β‐actin as a “loading” control (instead of GAPDH) in hypoxic studies, accordingly to a reported study.^19^

### 2.4 HIF‐1α transcriptional activity

Protein activity of HIF‐1α was measured using a commercially available HIF‐1α transcriptional factor assay kit (ab133104, abcam) according to the manufacturer's instructions.

### 2.5 Cell proliferation

Before cell viability assay using the Counting Kit‐8 (CCK‐8) method, BxPc‐3 cells (3.0 × 10^3^/100 μL/well) in a 96‐well culture plate were cultured overnight. After treatment at 0, 24, 48, 72 hours, 10 μL of CCK‐8 working solution was added to each well. Optical density (OD) values were recorded by a microplate reader (Bio‐Rad) at 450 nm.

### 2.6 Cell invasion assay

The ability of BxPc‐3 cells to invade Matrigel (80 μL, 356234, Corning) was determined using Transwell chambers (COSTAR, 3422). Briefly, 0.7 mL of RPMI‐1640 with 10% FBS was added to the lower chamber, whereas, BxPc‐3 cells (5.0 × 10^4^) in 0.2 mL of serum‐free RPMI‐1640 were added to the upper chamber. After 48 hours. Cells passing through the membrane were fixed with 4% of formaldehyde, dyed using crystal violet (0.05%), and counted using a light microscope (x100 magnification) in eight randomly selected fields.

### 2.7 Plasmids

DNA fragment encoding LOXL2 mRNA (AF117949.1) was amplified from human cDNA with the primers 5′‐CGGAATTCATGGAGGGCTACGTGGAGG‐3′ (forward) and 5′‐CGGGATCCTCAGGTAGCAGCCCCCCATG‐3′ (reverse), which was inserted into pLVX‐Puro (Clontech). The pLVX‐puro without LOXL2 expression was used as control vector. pLVX‐Puro‐LOXL2 (1000 ng), psPAX2 (100 ng), and pMD2G (900 ng) were cotransfected into 293T cells (ATCC) using Lipofectamine 2000 at 1:2 according to supplier's protocol (Invitrogen). About 1.5 μg of high‐titer recombinant lentiviruses with LOXL2 expression was transfected into BxPc‐3 cells using Lipofectamine 2000 reagent. After 48 hours transfection, transfected efficiency was assessed using RT‐PCR and Western blot analyses.

### 2.8 Xenograft model

Eighteen nude mice (Shanghai Laboratory Animal Company) were subcutaneously injected with BxPc‐3 cells (4 × 10^6^ cells_,_ 100 μL) and randomly divided into three groups: control group, salidroside (25 mg/kg) group, and salidroside (50 mg/kg) group. After tumors formation (approximately 1‐2 weeks later), tumor volume (mm^3^) was calculated every 3 days from 12th day to 39th day. When the tumor volume reaching 100 mm³ , mice in the control group was normal fed without any treatment, mice in two salidroside groups were intragastrically administrated with salidroside (B20504,YuanYe Biotechnology (Shanghai) Co., Ltd, Shanghai, China) at a dose of 25 mg/kg and 50 mg/kg, respectively. Three weeks later, all the mice were killed. Tumor weight were calculated and metastasis in lung was assessed using hemotoxylin and eosin (H&E) staining according to a reported study.^20^ We confirmed that the use of animal was approved by animal ethics committee of Shuguang Hospital affiliated to Shanghai University of Traditional Chinese Medicine.

### 2.9 TUNEL reaction

Cell apoptosis in mouse tumor tissue was detected using terminal‐deoxynucleotidyl transferase‐mediated dUTP nick end labeling (TUNEL) technology. Briefly, deparaffinized and hydrated tissue section (4‐7 μm) on slides was incubated with TUNEL working solution (Roche) at 37°C for 1 hour, then rinsed with phosphate‐buffered saline (PBS) (3 minutes × 3). Cell nucleus were double stained using DBA substrate kit (Long Island, Shanghai, China) followed by hematoxylin (BASO, Beijing, China). IMS image system (JRDUN, Shanghai, China) was used for visualization and analysis.

### 2.10 Statistical analysis

Each experiment was repeated at least three times, and data were expressed as mean ± standard error of the mean. Two‐tailed unpaired *t* test was used to compare between two groups, and one‐way analysis of variance (ANOVA) with post hoc Tukey's test was used to compare between two or more groups. *P* < 0.05 was statistically significant.

## 3 RESULTS

### 3.1 Salidroside suppressed the expression of *LOX* family genes

**Figure 1:**
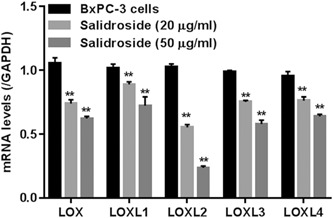
Effect of salidroside on expression of *LOX* family genes in BxPC‐3 cells. Normally cultured cells were treated with salidroside (20 and 50 μg/mL), and then mRNA levels of LOX and LOXL (‐1, ‐2, ‐3, and ‐4) were assessed, by RT‐PCR. GAPDH was used for normalization. **P < 0.01 vs BxPC‐3 cells treated with vehicle. mRNA, messenger RNA; RT‐PCR, reverse‐transcription polymerase chain reaction

BxPC‐3 cells were treated with 0, 20, 50 μg/mL of salidroside, after 24 hours, mRNA expression of *LOX* family genes (LOX as well as LOXL‐1, ‐2, ‐3, and ‐4) was measured by the RT‐PCR. As shown in Figure 1, salidroside (20 and 50 μg/mL) dose‐dependently inhibited *LOX* family genes with maximum effect being obtained on LOXL2 when compared with the BxPC‐3 normal control group (all *P* < .01).

### 3.2 Salidroside suppressed hypoxia‐associated HIF‐1α ant its target gene *LOXL2*

**Figure 2:**
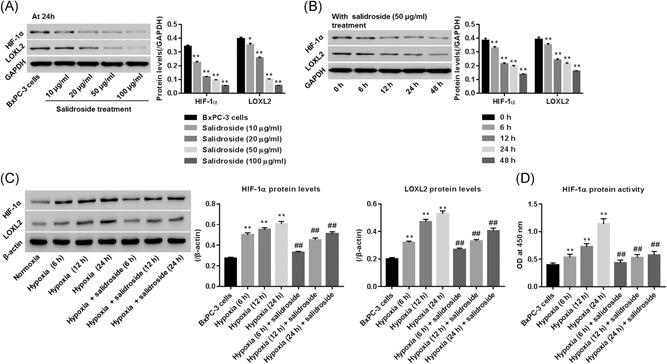
Effect of salidroside on expression of HIF‐1α and LOXL2 in BxPC‐3 cells cultured under normoxia and hypoxia. A, Cells were treated with salidroside (10, 20, 50, and 100 μg/mL), and then protein levels of HIF‐1α and LOXL2, normalized by GAPDH, were assessed using Western blot analysis at 24 hours. B, Cells were treated with salidroside (50 μg/mL), and then protein levels of HIF‐1α and LOXL2 were assessed at 0, 6, 12, 24, and 48 hours using Western blot analysis. C, Cells treated with salidroside (50 μg/mL) were exposure to hypoxia for 6, 12, and 24 hours, and then protein levels of HIF‐1α and LOXL2, normalized using β‐actin, were measured by Western blot analysis. D**,** protein activity of HIF‐1α, measured by an available bioassays method. **P < 0.01 vs BxPC‐3 cells cultured at normoxia without any treatment

BxPC‐3 cells were treated with 0, 10, 20, 50, and 100 μg/mL of salidroside, after 24 hours, protein expression of HIF‐1α and LOXL2 were measured by Western blot analysis. Figure 2A showed that salidroside significantly reduced HIF‐1α and LOXL2 in a dose‐dependent manner when compared with the BxPC‐3 normal control group (all *P* < .01). In this study, we chose 50 μg/mL as an ideal concentration for salidroside to treat BxPC‐3 cells. After treatment, protein levels of HIF‐1α and LOXL2 were determined at 0, 6, 12, 24, and 48 hours. Figure 2B showed that salidroside time‐dependently reduced the expression of HIF‐1α and LOXL2, and the significant effects were observed at 6, 12, 24, and 48 hours when compared with the BxPC‐3 normal control group.

We further studied the roles of salidroside in regulating HIF‐1α and LOXL2 in BxPC‐3 cells under hypoxia condition. Our data suggested that in comparison with normoxia, hypoxia time‐dependently enhanced the expression of HIF‐1α and LOXL2 and transcriptional activity of HIF‐1α. However, hypoxia‐induced the promotion of HIF‐1α and LOXL2 was significantly reversed and restored by salidroside to almost normal level at 6, 12, and 24 hours, respectively (Figure 2C and 2D).

### 3.3 Salidroside and KC7F2 inhibited proliferation and invasion of BxPC‐3 cells under hypoxia

**Figure 3:**
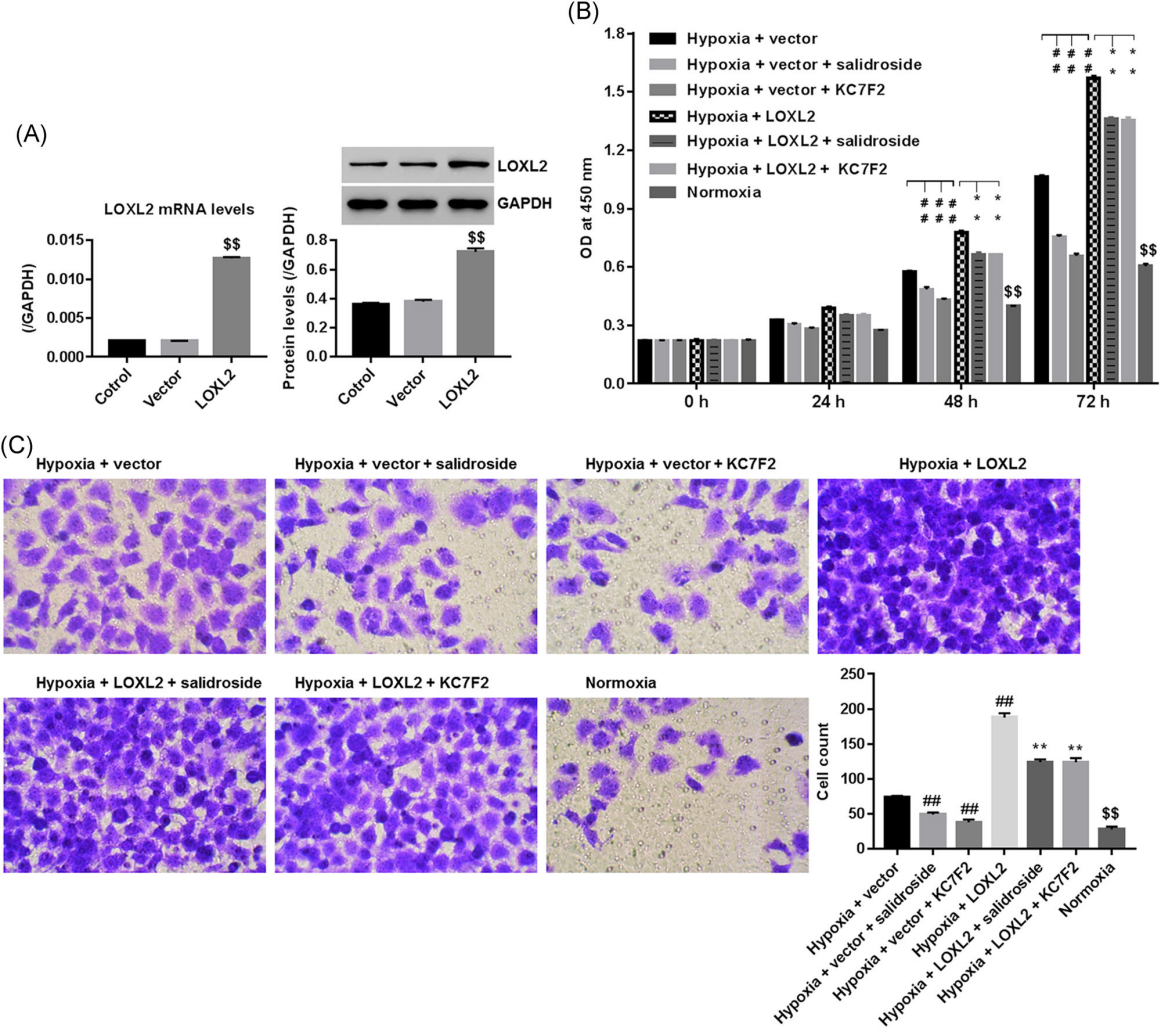
Roles of salidroside and KC7F2 in proliferative and invasive regulation of BxPC‐3 cells cultured under hypoxia. A, Significantly enhanced levels of LOXL2 mRNA and protein, demonstrating a successful establishment of LOXL2 overexpression within BxPC‐3 cells. Cells transfected with LOXL2 overexpression/control vector were treated with salidroside (50 μg/mL) or KC7F2 (50 μM). B, Cell proliferation at 0, 24, 48, and 72 hours, assessed using the CCK‐8 method. C, Cell invasion at 48 hours, assessed using Transwell chambers (x100 magnification). $$*P* < 0.01 vs normoxia; ##*P* < 0.01 vs hypoxia + vector; **P < 0.01 vs hypoxia + LOXL2. mRNA, messenger RNA

mRNA and protein levels of LOXL2 were significantly enhanced in cells transfected with lentiviruses expressing LOXL2 at 48 hours when compared with control vector (Figure 3A), suggesting a successful establishment of LOXL2 overexpression within BxPC‐3 cells. We further measured the changed expression of LOXL2 after different treatment at 48 hours. Our data showed that expression of LOXL2 under hypoxia was significantly enhanced by LOXL2 overexpression while reduced by both salidroside and KC7F2. Similar to 48 hours, the same change tread of LOXL2 expression was also obtained at 72, however, data at 72 hours has not shown in our present study.

To study the roles of salidroside on proliferation and invasion of BxPC‐3 cells under hypoxia, cells transfected with LOXL2 overexpression were cultured in hypoxia, and then exposed to 50 μg/mL of salidroside or 50 μM of KC7F2 (an HIF‐1α inhibitor, S7946, Sellck). Figure 3B and 3C suggested that hypoxia significantly enhanced proliferation and invasion of BxPC‐3 cells when compared with normoxia, and interestingly, hypoxia‐induced the promoted effect on BxPC‐3 cell proliferation and invasion was strongly augmented by LOXL2 overexpression. However, salidroside and KC7F2 resulted in the opposite, significantly repressing proliferation and invasion of BxPC‐3 cells with LOXL2 overexpression under hypoxia. Besides, as LOXL2 overexpression increased proliferation of BxPC‐3 cells by approximate 1.35‐fold at 48 hours, however, enhanced invasive ability of BxPC‐3 cells by approximate 2.50‐fold at 48 hours, substantiating the promoted effect of LOXL2 overexpression on cell invasion, which offset the increased in cell invasion caused by the promoted cell proliferation.

### 3.4 Salidroside and KC7F2 inhibited LOXL2, E‐cadherin, MMP2, and MMP9 in BxPC‐3 cells under hypoxia

**Figure 4:**
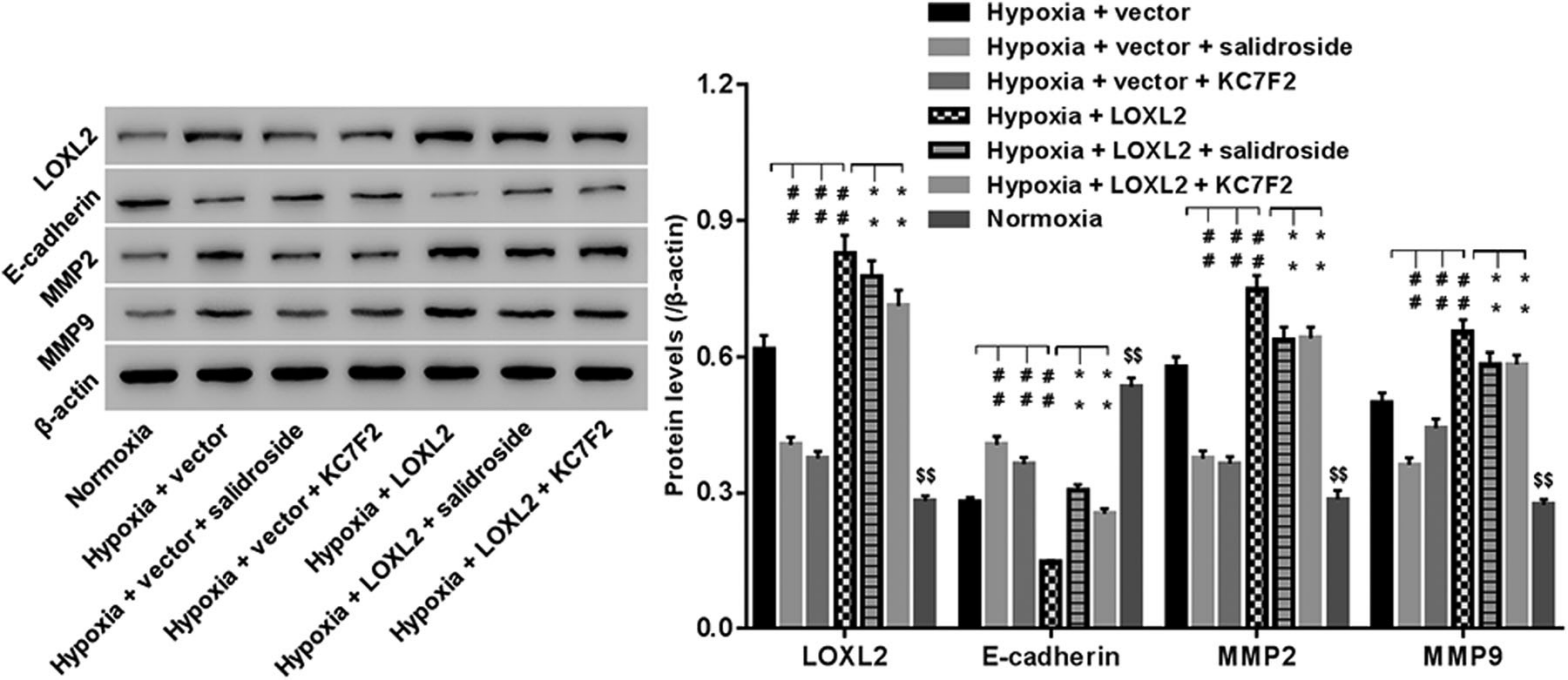
Roles of salidroside and KC7F2 in expression of LOXL2, E‐cadherin, MMP2, and MMP9 in BxPC‐3 cells under hypoxia. Cells transfected with LOXL2 overexpression/control vector were treated with salidroside (50 μg/mL) or KC7F2 (50 μM), and then protein levels of LOXL2, E‐cadherin, MMP2, and MMP9 were assessed at 48 hours. β‐actin was used for normalization. $$*P* < 0.01 vs normoxia; ##*P* < 0.01 vs hypoxia + vector; **P < 0.01 vs hypoxia + LOXL2

LOXL2, E‐cadherin, and MMP2/9 are involved in cancer cell invasion and metastasis. BxPC‐3 cells transfected with LOXL2/control vector were treated with salidroside or KC7F2 under hypoxia, and then protein levels of E‐cadherin, MMP2, and MMP9 were assessed, using Western blot analysis. Figure 4 demonstrated that hypoxia significantly increased LOXL2, MMP2, and MMP9, while decreased E‐cadherin when compared with normoxia, and the effects of hypoxia on those protein expression were strengthened by LOXL2 overexpression. However, hypoxia or LOXL2 overexpression‐induced changes in the expression of LOXL2, MMP2 and MMP9, and E‐cadherin were significantly reversed by additional treatment of salidroside or KC7F2.

### 3.5 Salidroside inhibited tumorigenicity of BxPC‐3 cells in nude mice

**Figure 5:**
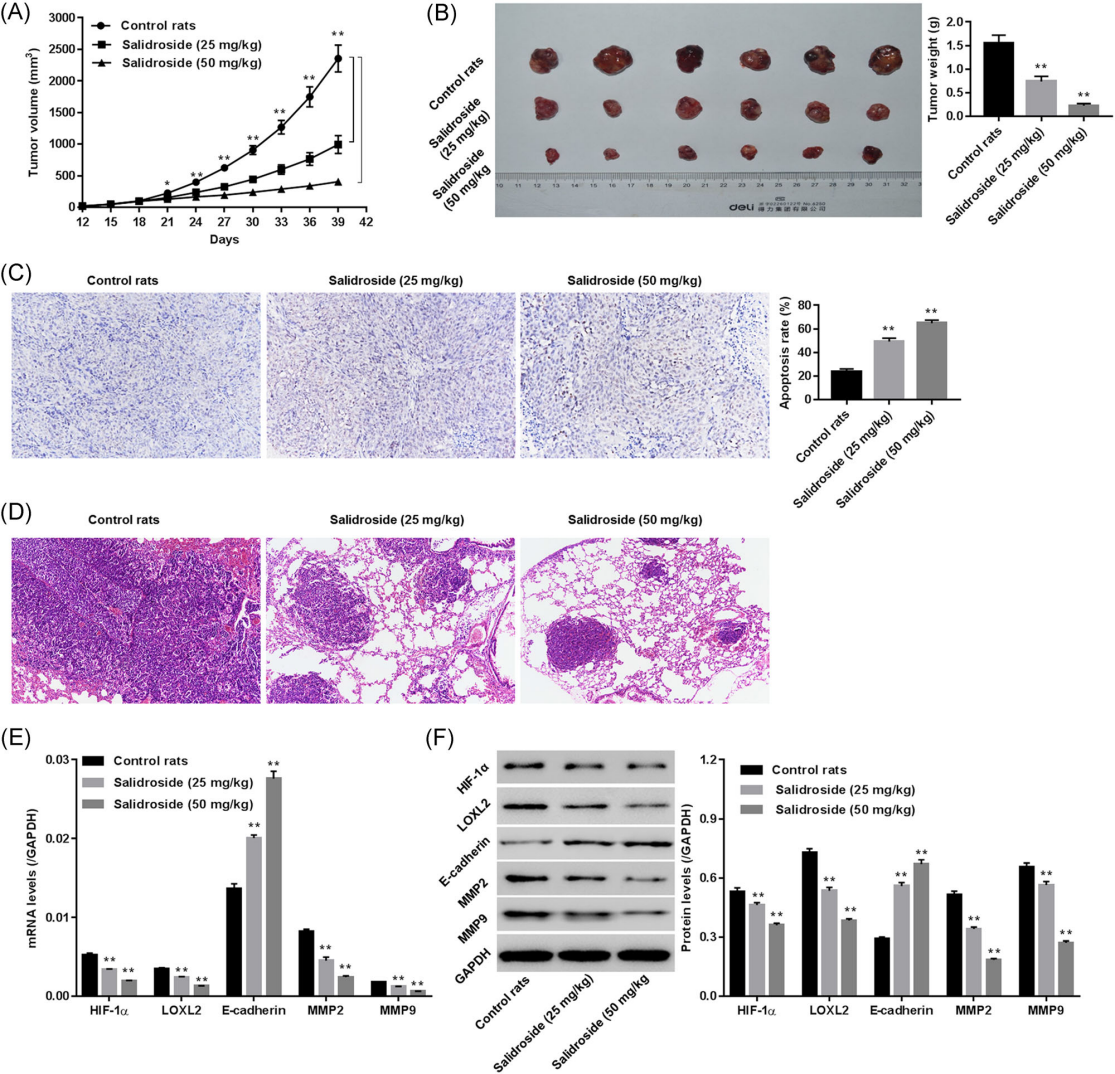
Salidroside inhibited tumorigenicity of BxPC‐3 cells in a Xenograft model. Nude mice were subcutaneously injected with BxPC‐3 cells (4 × 10^6^ cells_,_ 100 μL). Mice with tumor formation (100 mm³ ) were treated with vehicle (physiological saline), 25 mg/kg of salidroside, and 50 mg/kg of salidroside, respectively (*n* = 6 in each group). A, B, Tumor volume (mm^3^) measured from 12th to 39th day and tumor weight (g) calculated on 39th day. C, TUNEL results showing apoptosis in tumor tissue ( × 200). **D**, H&E staining showing metastatic lesions in lung tissue. E, F, Expression of LOXL2, E‐cadherin, MMP2, and MMP9 in tumor tissue. **P < 0.01 vs control rats. H&E, hemotoxylin and eosin; TUNEL, terminal‐deoxynucleotidyl transferase‐mediated nick end labeling

Evidence suggests that lung is one of the main sites for “metastases” manifestation when human BxPC‐3 cells are injected into nude mice.^21^ To study the roles of salidroside on tumor growth and metastasis, BxPC‐3 cell Xenograft was treated with salidroside (25 and 50 mg/kg). Our data suggested that salidroside dose‐dependently suppressed tumor volume and weight (Figure 5A and 5**B**), increased cell apoptosis (Figure 5C), reduced diffuse metastatic infiltration of BxPC‐3 cells in the lung tissues (Figure 5D), and significantly abrogated expression of HIF‐1α, LOXL2, MMP2, and MMP9, while enhanced E‐cadherin in tumor tissue (Figure 5E and 5**F**), suggesting the antigrowth and antimetastatic effect of salidroside in vivo.

## 4 DISCUSSION

LOXL2, a tumor promoter in PC progression, is a biomarker and therapeutic target in PC.^22^ Herein, we found that salidroside suppressed the expression of entire LOX family members, however, more effectively inhibited LOXL2 in a PC cell line (BxPC‐3) (Figure 1 **)**.

Hypoxia is a dominant regulator of HIF‐1α and LOXL2 (HIF‐1α directly transcriptional target) in the progression of various cancers.^23^ In PC, targeting HIF‐1α and LOXL2 are widely believed as therapeutic strategies to prevention in tumorigenesis of this disease. In hypoxia‐injured cardiomyocyte, salidroside is an inducer of HIF‐1α expression, and favors HIF‐1α translocation.^14^ In osteoporosis, salidroside inhibits HIF‐1α expression and translocation, however, increases the HIF‐1α transcriptional activity.^8^ Interestingly, our work showed that salidroside inhibited HIF‐1α expression and the transcriptional activity in hypoxia‐injured BxPC‐3 cells (Figure 2C and 2D), suggesting that salidroside may decrease HIF‐1α stability, and thereby influence HIF‐1α levels. Our work showed that salidroside was an inhibitor of HIF‐1α and LOXL2 in BxPC‐3 cells cultured both under normoxia or hypoxia (Figure 2).

In our present study, we confirmed that hypoxia promoted BxPC‐3 cell proliferation and invasion^24^ **(**Figure 3 **)**, and enhanced mobility property of BxPC‐3 cells through repressing E‐cadherin (a cell adhesion molecule and also a hallmark of EMT), while enhancing MMP2 and MMP9 expression (Figure 4). Those results suggested a severe carcinogenesis of hypoxia in BxPC‐3 cells. Salidroside inhibited proliferation and invasion of various cancer cell lines,^25^ which was further substantiated in BxPC‐3 cells under hypoxia (Figure 3). Besides, our data firstly suggested that salidroside inhibited BxPC‐3 cells to acquire migratory property through reducing MMP2 and MMP9, while increasing E‐cadherin under hypoxia (Figure 4). Interestingly, KC7F2 exerted the similar effect as that of salidroside, however, LOXL2 overexpression resulted the opposite. Notably, our data proved that salidroside and KC7F2 downregulated LOXL2, and meanwhile rescued the promoted influence of LOXL2 overexpression on proliferation and invasion of BxPC‐3 cells under hypoxia, confirming that downregulating LOXL2 was the mechanism, whereby, salidroside and KC7F2 delivered antitumor effect in hypoxia‐injured BxPC‐3 cells. Given the inhibitory roles of salidroside on regulating HIF‐1α expression, we can deduce that salidroside affected the proliferation and invasion of PC cells, likely by the HIF‐1α/LOXL2 pathway, which needed further substantiation in our laboratory.

HIF‐1α is a critical regulator of metastatic niche formation in the development of breast cancer (BrCa) probably through induction of LOXL2.^26^ Retarding HIF‐1α (knockdown/deficiency), or reducing hypoxia‐induced LOX family members in BrCa suppresses primary tumor growth in parallel with the metastasis of BrCa cells to the lungs**.** 26, 27 Moreover, a PC in vivo experiment suggested that salidroside suppressed tumor growth, increased cell apoptosis, inhibited the metastasis of BxPC‐3 cells to the lung, and reduced HIF‐1α, LOXL2, MMP2, and MMP9 while enhanced E‐cadherin in tumor tissue (Figure 5). These results substantiated the antitumor and antimetastatic effect of salidroside in PC in vivo, and the involvement of HIF‐1α, LOXL2 in this process.

Taken together these results, salidroside suppressed BxPC‐3 cell proliferation and invasion by downregulating MMP2 and MMP9 while upregulating E‐cadherin. Salidroside may be a therapeutic agent targeting HIF‐1α and LOXL2 in PC chemoprevention.

## CONFLICT OF INTERESTS

It has no conflict of interest exits in the submission of this manuscript, and manuscript is approved by all authors for publication.

## DATA AVAILABILITY STATEMENT

The datasets used and/or analyzed during the current study are available from the corresponding author on reasonable request.
